# Measurements by A LEAP-Based Virtual Glove for the Hand Rehabilitation

**DOI:** 10.3390/s18030834

**Published:** 2018-03-10

**Authors:** Giuseppe Placidi, Luigi Cinque, Matteo Polsinelli, Matteo Spezialetti

**Affiliations:** 1 A^2^VI_Lab, Department of Life, Health & Environmental Sciences, University of L’Aquila, Via Vetoio 1, 67100 Coppito, L’Aquila, Italy; matteo.polsinelli@student.univaq.it (M.P.); matteo.spezialetti@graduate.univaq.it (M.S.); 2 Department of Computer Science, Sapienza University, Via Salaria 113, Rome 00198, Italy; cinque@di.uniroma1.it

**Keywords:** Virtual Glove, LEAP motion controller, hand tracking, hand rehabilitation

## Abstract

Hand rehabilitation is fundamental after stroke or surgery. Traditional rehabilitation requires a therapist and implies high costs, stress for the patient, and subjective evaluation of the therapy effectiveness. Alternative approaches, based on mechanical and tracking-based gloves, can be really effective when used in virtual reality (VR) environments. Mechanical devices are often expensive, cumbersome, patient specific and hand specific, while tracking-based devices are not affected by these limitations but, especially if based on a single tracking sensor, could suffer from occlusions. In this paper, the implementation of a multi-sensors approach, the Virtual Glove (VG), based on the simultaneous use of two orthogonal LEAP motion controllers, is described. The VG is calibrated and static positioning measurements are compared with those collected with an accurate spatial positioning system. The positioning error is lower than 6 mm in a cylindrical region of interest of radius 10 cm and height 21 cm. Real-time hand tracking measurements are also performed, analysed and reported. Hand tracking measurements show that VG operated in real-time (60 fps), reduced occlusions, and managed two LEAP sensors correctly, without any temporal and spatial discontinuity when skipping from one sensor to the other. A video demonstrating the good performance of VG is also collected and presented in the Supplementary Materials. Results are promising but further work must be done to allow the calculation of the forces exerted by each finger when constrained by mechanical tools (e.g., peg-boards) and for reducing occlusions when grasping these tools. Although the VG is proposed for rehabilitation purposes, it could also be used for tele-operation of tools and robots, and for other VR applications.

## 1. Introduction

Hand rehabilitation is extremely important for recovering from post-stroke or post-surgery residual impairments and its effectiveness depends on frequency, duration and quality of the rehabilitation sessions [1]. Traditional rehabilitation requires a therapist for driving and controlling patients during sessions. Procedure effectiveness is evaluated subjectively by the therapist, basing on experience. In the last years, several automated (tele)rehabilitation gloves, based on mechanical devices or tracking sensors, have been presented [2,3,4,5,6,7,8,9,10]. These gloves allow the execution of therapy at home and rehabilitation effectiveness can be analytically calculated and summarized in numerical parameters, controlled by therapists through Internet. Moreover, these equipment can be easily interfaced with virtual reality (VR) environments [11], which have been proven to increase rehabilitation efficacy [12]. Mechanical devices are equipped with pressure sensors and pneumatic actuators for assisting and monitoring the hand movements and for applying forces to which the patient has to oppose [13,14]. However, they are expensive, cumbersome, patient specific (different patients cannot reuse the same system) and hand specific (the patient cannot use the same system indifferently with both hands). Tracking-based gloves consist of computer vision algorithms for the analysis and interpretation of videos from depth sensing sensors to calculate hand kinematics in real time [10,15,16,17,18,19]. Besides depth sensors, LEAP [20] is a small and low-cost hand 3D tracking device characterized by high-resolution and high-reactivity [21,22,23], used in VR [24], and has been recently presented and tested with success in the hand rehabilitation, with exercises designed in VR environments [25]. Despite the advantages of using LEAP with VR, a single sensor does not allow accurate quantitative evaluation of hand and fingers tracking in case of occlusions. The system proposed in [10] consisted on two orthogonal LEAPs designed to reduce occlusions and to improve objective hand-tracking evaluation. The two sensors were fixed to a wood support that maintained them orthogonal each other. The previous prototype was useful to test the robustness of each sensor, in presence of the other, to the potential infra-red interferences, to evaluate the maintenance of the maximum operative range of each sensor and, finally, to demonstrate the hand tracking idea. However, it was imprecise, due to the usage of raw VG support and positioning system, the non-optimal reciprocal positioning of the sensors, and the impossibility of performing a reciprocal calibration independent of the sensors measurements. This fact did not allow the evaluation of the intrinsic precision of the VG and to perform accurate, real-time quantitative hand tracking measurements. In this paper, we present a method for constructing an engineered version of the LEAP based VG, a technique for its accurate calibration and for collecting accurate positioning measurements and high-quality evaluation of positioning errors, specific of VG. Moreover, real-time experimental hand tracking measurements were collected (a video demonstrating its real-time performance and precision was also provided in the Supplementary Materials), presented and discussed.

## 2. Materials and Methods

### 2.1. System Design

**Figure 1:**
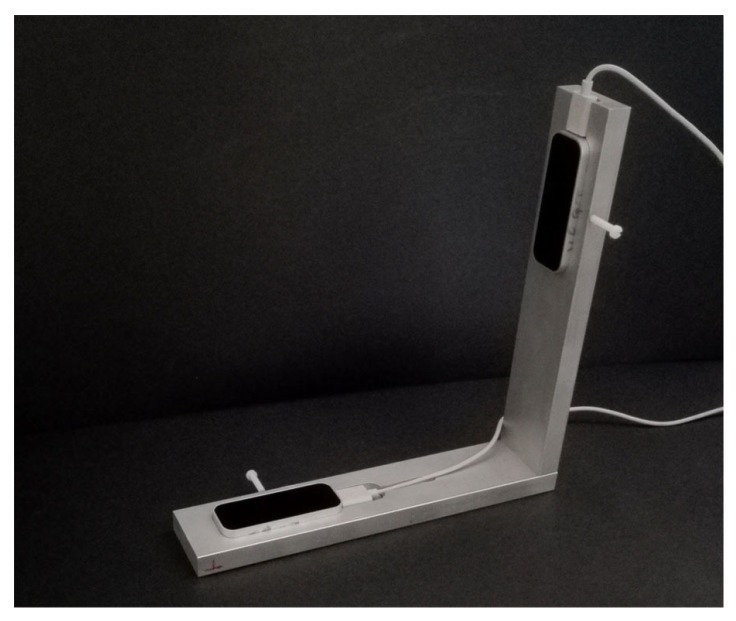
VG mounted on its aluminium support.

**Figure 2:**
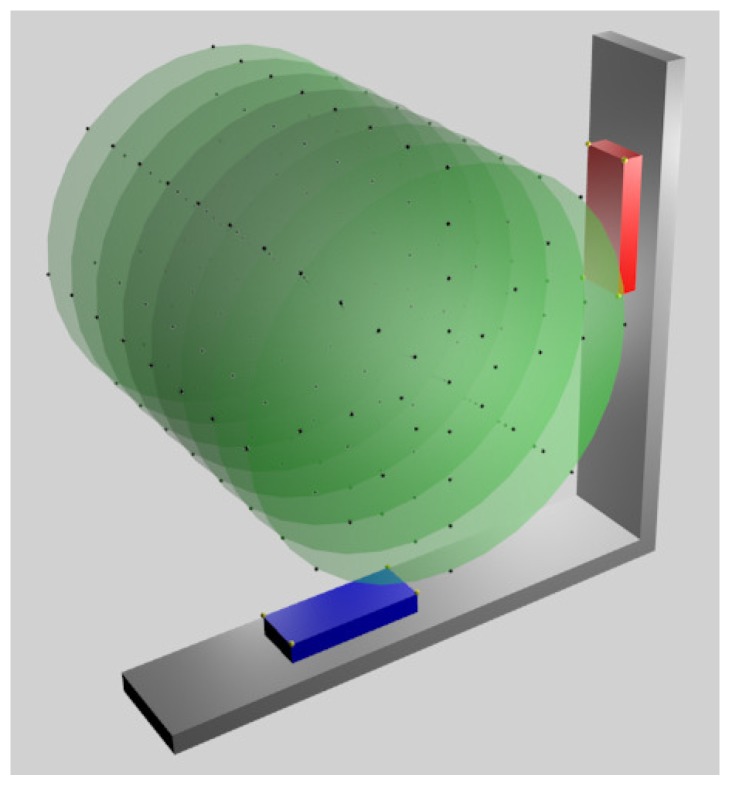
Representation of the measurements points inside a cylindrical green ROI, cut into circles spaced 3 cm from each other and of the points (represented in yellow) collected on the corners of the superior surface of each LEAP.

The VG system was designed to obtain simultaneous information from two LEAPs, orthogonally placed each other. The reason is that a single sensor is unable to compute with accuracy hand/fingers locations in the case of occlusions, that is, if the palm is approximately perpendicular to the sensor plane. The usage of two orthogonal sensors should ensure that at least one of them would maintain a favourable view of the hand. The VG support was realized in aluminium and lodges for the sensors, of the same shape of a LEAP cover base, were realized by using a numerical control mill (GUALDONI Mod. FU 80, 1995, Milan (Italy), spatial precision 0.01 mm). The LEAPs were fixed in position inside the lodges through plastic screws to avoid possible vibrations and movements (see Figure 1 for the assembly details). The centre of each LEAP was positioned at 18.5 cm from the internal part of the corner of the support: this was established for maximizing the signal in a region of interest (ROI) of radius 10 cm and height 21 cm (the cylinder axis was perpendicular to the plane of the VG support, as shown in Figure 2), while also reducing VG dimensions. In fact, by using the experience gathered in [10], we found that the direct infra-red radiation from one LEAP to the other was negligible and the positioning precision worsen with the distance from the sensors. The design and construction of the support was very accurate and the only movement necessary to overlap one LEAP to the other was a rotation of 90${}^{\circ }$ around the axis perpendicular to the plane of the support.

**Figure 3:**
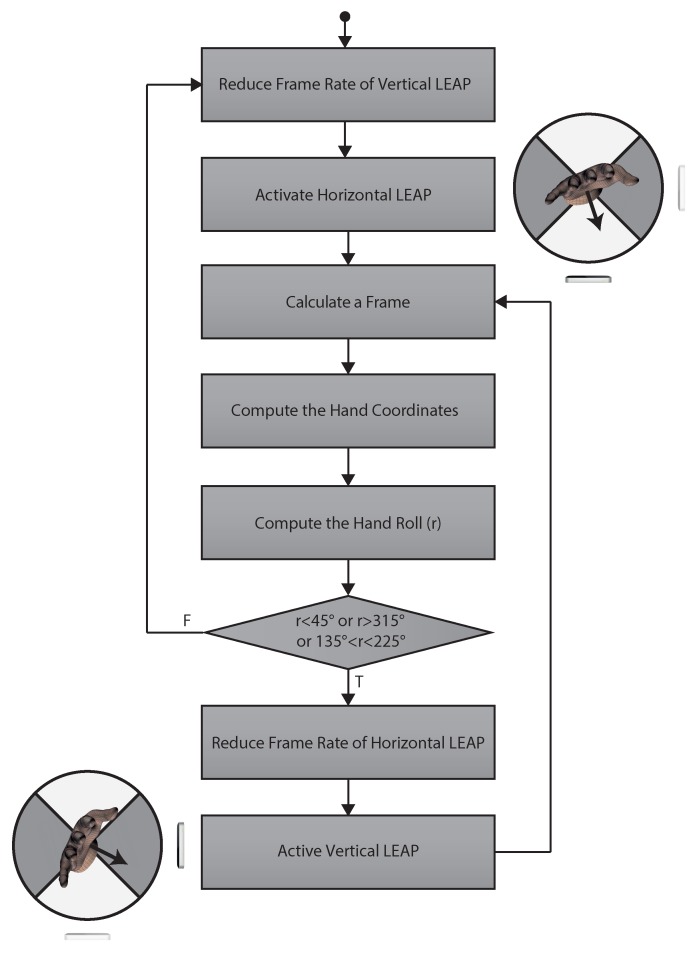
Flow chart of the tracking procedure: the hand was tracked by both sensors, the roll r with respect to the horizontal reference system was computed and used to determine the active sensor. The corresponding active sensor was graphically illustrated on the flow chart sides. The hand coordinates were always referred to the horizontal LEAP reference system.

One of the major issues was the simultaneous management of multiple instances of the LEAP since two sensors cannot be driven simultaneously by the same machine. This was solved using a virtual machine. The virtual machine (Slave) was installed on the physical machine (Master). One sensor was assigned to Master and the other to Slave, thus allowing each machine the instantiation of its own, single, driver. Data provided by both machines were rerouted towards a server (hosted on Master) to send data of both devices to clients running on Master. The hand tracking strategy for the proposed VG was based on mutual exclusion. The algorithm used for data acquisition, illustrated in Figure 3, was based on a control switching approach: both LEAPs acquired their own data stream, but only one sensor, depending on the rotation of the hand with respect to the horizontal LEAP reference system, was used to represent the hand and selected as “active” (the algorithm used for hand representation was that used internally by the LEAP). The palm vector **v**, perpendicular to the palm and provided by the sensor software, was used for computing the roll *r* of the hand, that is the angle between the *x* axis and the projection of the vector on the *x*–*y* plane, with respect to the horizontal LEAP reference system. If *r* was inside the interval 225${}^{\circ }$–315${}^{\circ }$ (the palm was facing downwards) or inside 45${}^{\circ }$–135${}^{\circ }$ (the palm was directed upwards), the horizontal LEAP was selected as “active” and the frame rate of the vertical sensor was reduced for saving computational resources), and vice-versa for the other LEAP. When vertical sensor was active, the server provided its coordinate change to the horizontal LEAP reference system. The server was also responsible of checking for the active LEAP, sending data to the client and, if needed, switching the status of the sensors. For using information from both sensors, their reciprocal position had to be computed with high accuracy through calibration.

### 2.2. Calibration

**Figure 4:**
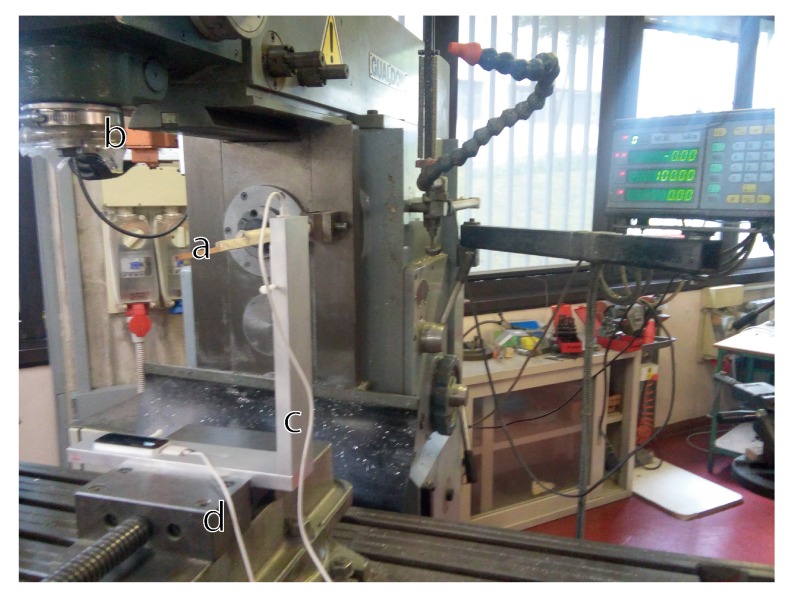
The mill equipped with the three axes movement system. The stick (**a**) was fixed to the static structure of the mill also hosting the mill tool (**b**). The VG structure (**c**) was secured by a grip to the three axes moving block of the mill (**d**), controlled numerically. The stick was used both to measure the LEAPs position with respect to the mill reference system and to collect spatial measurements inside the ROI.

The positioning system of the mill, used for drilling the VG support, was also used for collecting spatial measurements of the tip of a stick rigidly fixed to the mill (Figure 4). The VG support, fixed to the three axes moving system of the mill and oriented to allow the internal reference system of each LEAP to have the same directions as the mill, was moved along the three axes with a precision of 0.01 mm while the stick was maintained still. In that way, it was possible to collect measurements inside the defined region of interest. The first measurements were those regarding the vertexes of the superior surface of the LEAPs (Figure 2, indicated by small yellow spheres). This allowed evaluating LEAPs position/orientation with respect to the mill reference system (spatial calibration was obtained and positioning/orientation differences from the awaited were easily estimated and corrected).

The transformation matrices were derived from these measurements, separately for each of the two sensors, through a Singular Value Decomposition (SVD) [10,26,27,28] in homogeneous coordinates, with respect to the mill reference system:

(1) $$W_{horizontal}=\left[\begin{array}{cccc} 1.0 & 0.0 & 3.8\times 10^{-4} & 1.2\\ 0.0 & 1.0 & 0.0 & -181.1\\ -3.8\times 10^{-4} & 0.0 & 1.0 & -4.8\\ 0.0 & 0.0 & 0.0 & 1.0 \end{array}\right]$$

(2) $$W_{vertical}=\left[\begin{array}{cccc} 0.0 & -1.0 & 0.0 & 171.7\\ 1.0 & 0.0 & 3.2\times 10^{-4} & -6.6\\ -3.2\times 10^{-4} & 0.0 & 1.0 & -4.9\\ 0.0 & 0.0 & 0.0 & 1.0 \end{array}\right]$$

However, the previous matrices just considered the external spatial displacement between LEAPs and not “logical” transformations due to internal differences between LEAPs and between each of their reference systems with respect to the mill reference system. To include these effects into the previous matrices, a series of spatial measurements with the horizontal LEAP were collected and then repeated on the same points with the vertical LEAP mounted in the horizontal box. In this way, effects of matrix transformation were excluded and differences were just due to internal constructive discrepancies between sensors. A total of 264 measurements (Figure 2) were collected on the surface of concentric cylinders (radii equal to: 0 cm, 2.5 cm, 5 cm, 7.5 cm and 10 cm; sampling angles: 0${}^{\circ }$, 45${}^{\circ }$, 90${}^{\circ }$, 135${}^{\circ }$, 180${}^{\circ }$, 225${}^{\circ }$, 270${}^{\circ }$, and 315${}^{\circ }$) with the axis oriented along the *z*-axis of the LEAPs (along *z*, a total of 8 slices, 3 cm apart, were measured around the centre of the VG system for a total length of 21 cm). In addition, these data were analysed through SVD to find the resulting transformation matrices, one for each LEAP. The transformation between the coordinate system of each LEAP was obtained and the mill coordinate system was simply an axis translation (no distortions or scaling factors occurred and just negligible fluctuations, due to measurement noise, to the upper left region of the matrix were present). The obtained translation matrices were added to transformation matrices in Equations (1) and (2) (before adding the contribution obtained for the vertical LEAP, it was first converted by using the inverse of matrix in Equation (2)) to obtain the final modified transformation matrices for the horizontal and vertical sensors, respectively:

(3) $$W_{horizontal}=\left[\begin{array}{cccc} 1.0 & 0.0 & 3.8\times 10^{-4} & 0.4\\ 0.0 & 1.0 & 0.0 & -189.6\\ -3.8\times 10^{-4} & 0.0 & 1.0 & 14.4\\ 0.0 & 0.0 & 0.0 & 1.0 \end{array}\right]$$

(4) $$W_{vertical}=\left[\begin{array}{cccc} 0.0 & -1.0 & 0.0 & 179.1\\ 1.0 & 0.0 & 3.2\times 10^{-4} & -1.0\\ -3.2\times 10^{-4} & 0.0 & 1.0 & 2,9\\ 0.0 & 0.0 & 0.0 & 1.0 \end{array}\right]$$

The described calibration procedure was made for maintaining the accurate transformation derived by spatial measurements while also considering the translation obtained by measurements collected with the sensors (we forced the step across LEAP measurements for including internal differences between LEAPs). No scaling effect was observed. The transformation matrices in Equations (3) and (4) were used for transforming data from both LEAPs to the mill reference system. It is important to note that calibration, being it done to define reciprocal position/orientation between sensors and not regarding the object to be tracked, has been done when VG is constructed, and it is completely independent of the object to be tracked.

## 3. Results

### 3.1. Static Measurements

**Figure 5:**
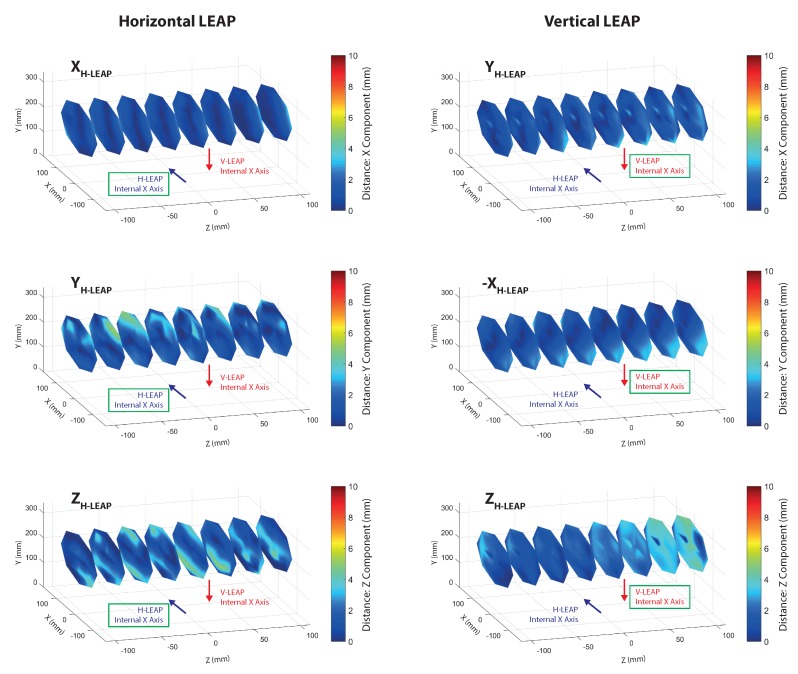
Distance between points measured by the mill positioning system and by each of the LEAPs along different axes after calibration. Since measurements were referred to the mill axes, the original LEAP axes were reported inside the graphs to allow a direct comparison between corresponding axes of both sensors. The position of the internal *x* axis of each LEAP was also reported. The green box indicated the “active” LEAP.

**Figure 6:**
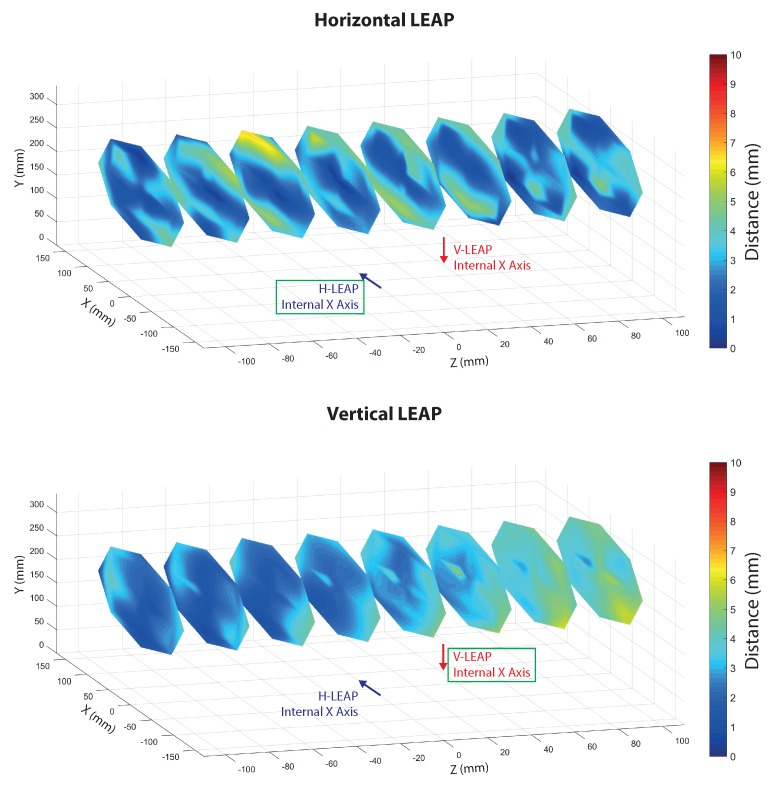
Global distances calculated from data of Figure 5 both for H-LEAP (upper panel) and for V-LEAP (lower panel).

Spatial characterization of the VG was performed by collecting measurements on the same positions used for calibration but with the LEAPs in their definitive positions of the aluminium support (as in Figure 2). Three types of data were collected: spatial information (stick tip position given by the mill positioning system) and data collected by each LEAP. LEAP data were transformed and reported to the world coordinate system (the mill reference system) and the absolute distances between points measured by each LEAP and the corresponding spatial measurements collected by the mill were calculated separately for each axis (Figure 5) and globally (Figure 6). The sequence of measurements was collected following trajectories aimed at optimizing the movements of the mill. The sampled measurements were interpolated to allow continuous graphic representations and improve readability. The reported data show that the error was lower than 6 mm in the considered ROI for both LEAPs, although some discontinuities, giving raise to particular “diamond-shape” figures, were present. The number of discontinuities was greater in the horizontal LEAP (H-LEAP) than in the vertical LEAP (V-LEAP), thus resulting in a less regular behaviour of H-LEAP with respect to V-LEAP. In V-LEAP, a coherent increment of the error along the axis of the ROI was present but, due to its coherence, it was probably due to external infra-red interferences, probably due to the presence of a window close to the position of the mill (see Figure 4). Average distance (error), standard deviation and maximum distance were calculated and reported in Table 1 separately for each LEAP. Despite the higher instability of H-LEAP and the coherent disturbance of V-LEAP, their maximum error, obtained along the *z* axis (that could be explained with the difficulty of the internal software of the sensor to track the tip of the stick along its long axis), remained below 6 mm. Since our scope was to identify finger tips and joints whose dimensions, for an adult person, are normally above 1 cm (that could be used as a maximum tolerable value for the error), the obtained results were good.

### 3.2. Hand Tracking Measurements

**Figure 7:**
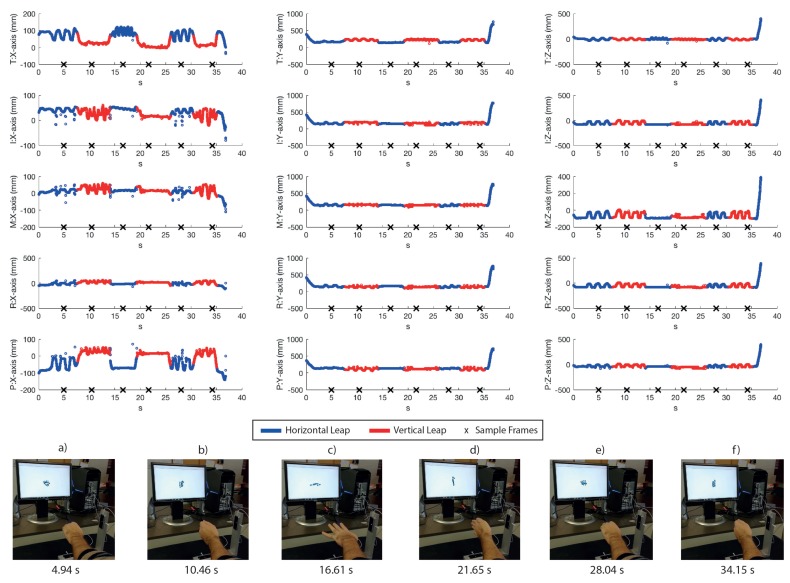
Real time VG tracking for each of the finger tips movements (T: thumb; I: index; M: middle; R: ring finger; and P: pinkie), for each of the three axes, registered for 37 s. In blue is indicated the horizontal LEAP signal used by VG and in red that of the vertical LEAP. In the low part, some snapshots, corresponding to the time instant indicated by crosses along the time axis, of an external video are reported for indicating the current hand orientation and fingers location and the corresponding visual model represented on a computer screen.

**Figure 8:**
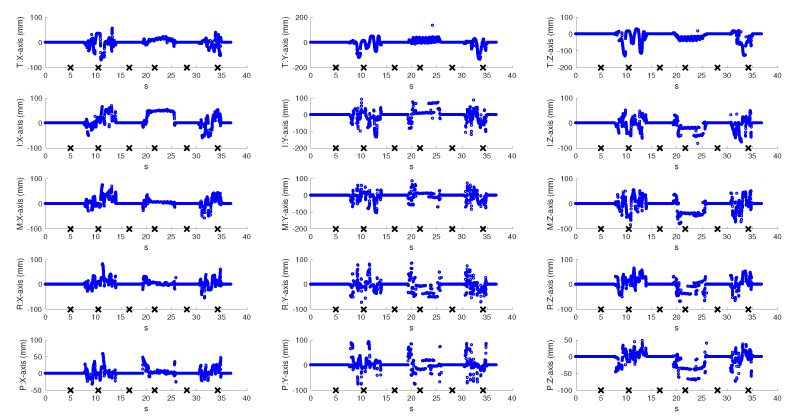
Differences between VG tracking measurements and horizontal LEAP measurements for each finger (T: thumb; I: index; M: middle; R: ring finger; and P: pinkie) and for each axis.

**Figure 9:**
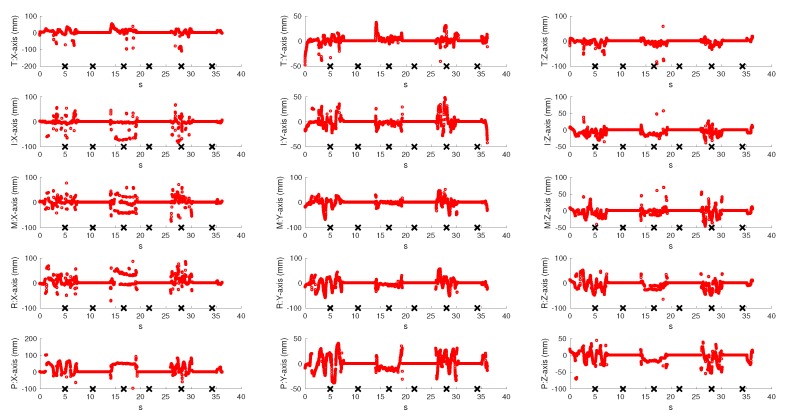
Differences between VG tracking measurements and vertical LEAP measurements for each finger (T: thumb; I: index; M: middle; R: ring finger; and P: pinkie) and for each axis.

To evaluate the real-time behaviour of the VG system, a left hand tracking sequence of 37 s, recording continuous palm rotations and fingers movements, was collected. It is important to note that we could have also performed a right hand tracking without any modification of the system: the only requirement would have been the insertion of the hand from the other side of the VG in order to ensure the view of the palm of the hand to both sensors. In fact, VG is completely independent of the hand inserted into it (left/right) and of its shape or size (this means that hands of different persons could be tracked by VG without any modification). The palm remained still for about 5 s and then performed fast rotations of 90${}^{\circ }$, with the first orientation parallel to the horizontal LEAP. Fingers were continuously moved and some positions were chosen to produce occlusions for at least one of the LEAPs. For example, fingers were moved back and forth on the palm plane by using the abduction/adduction movements of the first joint (e.g., spreading fingers apart), flexed to form fist, or completely extended. The resulting coordinates of all joints and of the palm were recorded for both LEAPs and for the VG system. Figure 7 shows the coordinates of the fingertips measured by the VG at 60 fps (to avoid confusion, just fingertips were shown, though all joints were tracked). Measurements collected with H-LEAP are indicated in blue and those collected with V-LEAP are in red. An external video (provided in the Supplementary Materials), synchronized with the acquisition/tracking process, was also collected to show how the hand was moving, to capture simultaneously the hand movements and model reconstruction/representation on a computer screen, to verify the fluidity of the movements and the absence of discontinuities when VG switched from one sensor to the other. Some snapshots of the video, presented in the lower part of Figure 7a–f, correspond to the crosses reported on the time axis, in particular: images in Figure 7a,b were collected while the hand was repeatedly open and closed by using all fingers, though along perpendicular directions; in Figure 7c,d, the hand was open and fingers were continuously abducted/adducted, but along perpendicular directions; Figure 7e,f repeated Figure 7a,b, respectively, to demonstrate measurement repeatability. Tails of the recorded hand movements reported in Figure 7 registered the phases of insertion and removal of the hand inside the VG. Measurements contained occlusions: for example, in Figure 7a, all the tips, except thumb and index, were occluded to V-LEAP and, in Figure 7d, all the tips were occluded to H-LEAP. In these cases, VG used measurements from one LEAP by excluding the other in a switching way. Figure 7, supported by the external video, also shows that, though switching from one LEAP to the other, the fluidity of the movements of the hand model represented on the computer screen was maintained without discontinuities, thus demonstrating the good accuracy of VG calibration. Moreover, the resulting VG frame rate, 60 fpm, demonstrated the maintenance of an optimal computational performance (each LEAP, separately, could be driven at 100 fpm), since for hand tracking rehabilitation purposes 20–25 fpm would be sufficient [25]. Figure 8 and Figure 9 reported the differences between the collected VG signals and those collected by each LEAP: obviously, differences were zero when VG used the signal corresponding to the active LEAP; some values were very high; and, due to occlusions, some values were highly spurious (high-frequency oscillations occurred). When a LEAP is unable to really “see” a point, in the case of occlusions, it tends to estimate its location, which can result very far from the real one. Moreover, the resulting estimated location can change rapidly with time as new frames arrived. Data from Figure 7, Figure 8 and Figure 9 are very useful to calculate the number of unmatched data (outliers) between the VG and each of the LEAP sensors.

To this aim, it was necessary to construct the reference tracking trajectories of the VG, the averaged VG (AVG), in which outliers were completely absent. The AVG was obtained by filtering out the frequency content above 25 Hz (that is 25 fpm, see above) from the tracking signals, in the Fourier space. The rationale behind this choice is that movements occurring faster than 1/25 s are considered unnatural, hence outliers. Differences between AVG and H-LEAP and between AVG and V-LEAP, respectively, were calculated (to allow a direct comparison with the reported data, the operation was applied just to the samples in Figure 7, Figure 8 and Figure 9, referred to fingertips, although it could be easily extended also to the other joints). A given sample was considered an outlier if at least one of its coordinates varied of more than 1 cm (the tolerance value, see above) from the value given by VG. The number of outliers was 3217 for H-LEAP and 2991 for V-LEAP, respectively. Regarding the total tracked points, 11,100, VG allowed saving about 29% of outliers with respect to simply using H-LEAP and about 27% with respect to simply using V-LEAP (the different proportions depended on the position of the hand with respect to VG and on the time it remained in a favourable position with the corresponding sensor). This means that, for the unfavourable positions of the hand with respect to the sensors, more than 55% of the points singularly tracked were outliers (since fingertips are the most external joints, it could be argued that this percentage would increase for intermediate joints). This fact justify the usage of two orthogonal LEAPs for implementing a VG for rehabilitation purposes. However, residual outliers were still present also by using VG, as shown in Figure 7. To calculate their number, the difference between AVG and VG was found, and outliers calculated as described above (in this case, a residual outlier was considered a VG tracking error). The residual number of outliers in VG was 177, about 1.5% of the whole tracked points and less than 8% with respect the number of occlusions by using a single LEAP sensor. As can be observed in Figure 7, residual outliers mainly occurred when using H-LEAP. They were probably due to local occlusions, occurring for points where also the favourable LEAP was obscured. However, the proposed tracking sequence showed that analogous movements were also performed with V-LEAP, but with a very low frequency of outliers (a possible explanation could be the lower stability of H-LEAP with respect to V-LEAP). Moreover, residual VG outliers occurred in two fo the six skips between LEAPs in Figure 7, thus confirming that orientation angles close to the border values could be critical for both LEAPs.

However, it is important to note that the number of residual outliers in VG was very low and that they could be completely corrected by filtering the collected tracking signal at 25 fps, as done for calculating AVG, without loss of temporal precision. Moreover, a further gain would be obtained by merging data from both LEAPs and not just using one LEAP at once: this could be particularly important for reducing outliers at border orientation angles.

In fact, the above data showed that some tips and positions were also visible by the unused LEAP, although from a different perspective (due to the volumetric shape of the fingertips, the two LEAPs “saw” different faces of the same object, located in different positions: this is the reason of not completely zero differences).

In summary, it can be argued that:

1. The accuracy and the fluidity of the tracking process were adequate and the change of perspective did not produce discontinuities or other appreciable effects.
2. The hand was correctly tracked also for positions that would be critical for a single LEAP scenario.
3. From the presented measurements, it was impossible to quantify exactly the positioning error (although it can be reasonably argued that it would be no worse than that reported in Table 1).

## 4. Discussion

The procedure for assembling a multiple-sensor VG for real time hand tracking, based on the use of two orthogonal LEAP sensors, and its calibration was illustrated. The error of the system, when collecting small objects (the dimension of the stick was about 5 mm × 5 mm) was lower than 6 mm in the considered ROI, thus making the proposed system well suitable for accurate hand tracking measurements. Hand tracking tests confirmed that: real-time hand model reconstruction was possible when using two orthogonal LEAPs; the algorithm used by VG switched correctly from one sensor to the other; no discontinuities were evident and calibration was effective; and most of the occlusions, highly frequent when a moving hand is tracked by a single LEAP, were eliminated, which could be particularly important when tracking of the hand is done while grasping a tool. Differences in location estimation remained when observing hand joints from different perspectives caused by joints volume. However, the obtained tracking resolution was good both spatially and in time, thus suggesting that, besides rehabilitation, VG paradigm could be also effectively employed in other applications, such as tele-operation of tools and robots. Next developments will regard the implementation of:

1. an efficient strategy for merging data coming from both sensors, in substitution of the mutual exclusion strategy, for further reducing occlusions while solving the problem, mentioned above, of the differences in localizing the same joint from different perspectives;
2. a numerical hand model, similar to that used in [5], to be associated to the virtual representation of the hand and to be used, beside registering movements, also for calculating forces and efforts exerted by each finger and by the whole hand, which considers the effects of gravity (these calculations, being particularly cumbersome and really interesting just for therapists, could be implemented in an off-line mode);
3. an efficient strategy for further reducing occlusions in the numerical hand model based both on the constraints between hand joints, joint angles and efficient temporal filtering (this would improve accuracy of dynamic parameters calculation);
4. a framework for developing rehabilitation tasks associated with virtual environments and for analysing numerical rehabilitation data and therapy outcomes;
5. a set of calibrated tools, mainly based on transparent springs to reduce occlusions with respect to the hand joints and interference with the LEAP sensors, to be used during rehabilitation exercises for applying resistance to motion.

Finally, we aim at testing the VG on patients under the supervision of specialists for evaluating its efficacy.

## Figures and Tables

**Table 1:** Average, standard deviation and maximum value of the distance, in mm, along each axis and in the space, of data reported in Figure 5.

Distance (mm)		H-LEAP	V-LEAP
*X* Axis	Average	0.8	1.3
	Standard Deviation	0.8	0.9
	Maximum	3.3	3.9
*Y* Axis	Average	1.4	1.4
	Standard Deviation	1.2	1.0
	Maximum	4.7	3.8
*Z* Axis	Average	1.7	2.0
	Standard Deviation	1.4	1.1
	Maximum	4.6	4.6
3D	Average	2.8	3.0
	Standard Deviation	1.4	1.1
	Maximum	6.6	6.0

## Supplementary Materials

The following are available online at http://www.mdpi.com/1424-8220/18/3/834/s1.
